# On the Defence: UK cultural narratives of mistrust between energy users and providers

**DOI:** 10.3934/publichealth.2018.1.31

**Published:** 2018-03-13

**Authors:** Cathy Bailey, Philip Hodgson

**Affiliations:** Health and Life Sciences, Northumbria University, Newcastle upon Tyne, NE7 7XA, UK

**Keywords:** energy services, older people, cultural narratives

## Abstract

In general, households rely on energy providers to supply essential energy services such as gas and electricity. It seems reasonable to assume that it is mutually beneficial to have a customer and supplier relationship invested in trust. Key findings from the qualitative evaluation findings of a UK Comic Relief-funded energy services and managing money better programme, suggest that the programme's effectiveness was strongly affected by negative narratives about energy suppliers. Such narratives, rooted in feelings of being labelled a ‘cheat’ or incapable of sorting their own affairs on one side and views of energy providers being exploitative and profit-hungry on the other, engendered a common, oppositional ‘united against them’ culture, built on reciprocal mistrust and disrespect. This analysis is not unique to our research, as nationally, at least and within the last decade, there has been a decline in public trust of energy providers, with a suggestion that profit has come before people. The 3-year evaluation carried out by Northumbria University, UK with the research led by a North East England registered credit union and social landlord, assessed the quality of life impacts of a face-to-face energy advice service. Expert Energy Advisors offered free home visits and gave people aged 50 and over the tools to reduce and manage energy usage, question energy companies about tariff terms and conditions and ensure maximum take up of benefit entitlements. Whilst findings point to positive health and social benefits, including reducing high anxiety about unmanageable bills, being able to question and challenge energy providers ‘high’ bills and tariffs and passing on such skills to others, there remained a ‘taken-for-granted’ mistrust of energy providers. We argue that for public good to come from public health research, we need to understand and appropriately address the roots of such cultural narratives.

## 1. Introduction

Much has been written about how we communicate through stories or narratives. From within public health and at least since the 1980s, there has been acknowledgement that narrative can be a fundamental influencing tool, used by social, political, health and social care institutions, for example, to share health information with us, the public [1]. Such narrative moves away from a paradigmatic way of knowing, with its verifiable facts and empirical truisms [2], which it could be argued has underscored traditional public health communication. Rather, narrative captures culturally-specific and complex social constructions and meanings ascribed to public health matters [3].

More recently and in relation to cultural attitudes to public health information, there has been interest in ‘narrative change’. Jones & McBeth [4] suggest that from within policy theory, narrative is still hard to pin down and that language slippage can also be a problem, with ‘narrative change’, for example, being used to also mean ‘attitude change’ and ‘culture change’. Davidson [5] of the US Open Society Public Health Program says that “narratives are important in guiding individual beliefs and decision making”, and that they should thus play a significant role in policy processes. He cites the work of Haidt [6] and Kahneman [7] and others to assert that human decision making is based on our ability to think automatically, socially and with mental models. We do not just think rationally.

Davidson [5] also introduces the work of Fisher [8] to contend that the rational paradigm of human decision making, that is argument based on ‘evidence’, is limited and does not capture ‘everyday argument’. Rather, the narrative paradigm, the ‘storytelling human’, trades in ‘good reason’ and ‘cultural signs and symbols’ that are influenced by our biographies, personalities, and cultures, with judgement perhaps based on whether competing narratives, or challenges to our narratives (‘narrative change’), ‘ring true’ or are probable [9]. In particular, Davidson [5] argues that making space for a narrative rather than a rational paradigm opens up *public* health decision making to the public. So for example, rationally-formed decisions, made by ‘technical experts’ about health care access, or allocation, or timeliness and duration of provision, may also be considered by us the public in terms of our trust in the experts, our culturally-infused health beliefs, and/or our experience of public health bodies and services.

Much of this fits with socio-ecological and holistic models of health behaviours [10] in that it is acknowledged that in order to effect positive public heath behavioural change, there needs to be a situated social, cultural and economic understanding of multiple perspectives, from individual to interpersonal, and from community to organisational and public policy, contexts. From a cultural perspective, and in some ways to counter the postcolonial transfer and imposition of predominantly Western health promotion and disease prevention programmes into very different cultural settings, there has also been the development of socio-ecological models such as PEN-3 [11] that locate identity at the intersection of culture and health. Public health interventions are planned at the local level and with some understanding of individual and community perceptions, enablers and nurturers (PEN). Such a model challenges a public health practitioner or researcher to start with local, individual perceptions (knowledge, belief, attitudes, motivation), rather than ‘needs’ identified by the technical experts, to consider the reality of what is or isn't available in relation to public health services and supports (what enables someone to make positive health changes) and how significant others (family, peers, employers, community leaders) may use a cultural lens that endorses ‘healthy’ or not so ‘healthy’ behaviours.

Moreover from within a social model of health, it is critical to include wider determinants of health such as the lack of transport to get to a health service, or a perceived lack of state financial assistance, when a basic need such as an energy supply to keep warm, to cook and to ‘power’ so much of daily life, becomes too costly or circumstances change abruptly. Faced with what might be viewed as a ‘rigid’ energy supplier with inflexible penalties resulting in escalating bills, and if wider family, neighbours, or colleagues are facing similar circumstances, it is likely that both punitive provider and reckless user narratives, with some gradients in between, may become a ‘narrative truth’. This may be the case even when faced with a challenging ‘other’ narrative that demonstrates a ‘flexible’ energy supplier or ‘prudent user’, because the changed narrative doesn't ‘ring true’.

As public health researchers we often work with individuals and communities who remind us that narratives are dynamic, plural and may be embedded in institutions, work practices and a myriad of competing cultures. Moreover we become *aware* of such narratives, they may not be directly spoken of but rather, to echo Lepselter [12], their assumptions become the culture in which people act out everyday activities, and their symbols of action, word and deed are not examined or questioned. They just are.

Drawing on the qualitative evaluation findings from a UK Comic Relief-funded energy service and managing money better programme, this paper explores ubiquitous local cultural narratives, arising from some personal, negative, customer experiences with energy providers. Such narratives, rooted in feelings of being labelled a ‘cheat’ or incapable of sorting their own affairs on one side and views of energy providers being exploitative and profit-hungry on the other, engendered a common, oppositional ‘united against them’ culture, built on reciprocal mistrust and disrespect. This analysis is not unique to our research, as nationally, at least within the last decade, there has been a decline in public trust of energy providers, with a suggestion that profit has come before people [13]–[15]. In the next section we consider this mistrust in relation to public health.

## 2. Domestic energy provision in the UK and public health context

Energy supply, via gas and electricity, needs to be safe, reliable and affordable. Reports tell us that customers trust suppliers that can give the best outcomes that serve their interests [16]. In the UK there are some 5,000 community energy groups providing over 60 MW of renewable generating capacity, and it is suggested that such schemes benefit local communities by investing in energy efficiency to reduce energy bills, create jobs and provide advice to those in fuel poverty. However government reductions in wind and solar power and changes to funding initiatives, have left the future of such groups uncertain. There are some new municipal energy providers that are trying to provide and maintain lower prices for their customers but most of the UK population is still dependent on the ‘big six’ utility companies to provide their gas and electricity.

The Energy Companies Satisfaction Survey 2017 [15] results demonstrate that the smaller companies that do exist, fare better than the UK's Big Six energy suppliers. For example, one of the latter received a customer score of just 44%, compared with one of the former's 78% customer score. The survey asked 8,917 energy customers to rate their energy provider on a range of measures, including customer service, whether it deals with complaints, whether it is value for money, the clarity and accuracy of its bills, and how their supplier helps them save energy [15]. Results from earlier polling [17] and cited within Carbon Brief's, Public Opinion, online summary [14], suggest that energy prices increasing in the face of continued provider profit and lack of industry regulation, leads to overall public mistrust in energy providers. An earlier 2013 report, [13] on UK utilities provision, contends that given the essential nature of the need for a continuous utility supply, there should be a provider-customer relationship that is built on mutual trust and confidence. However they noted the then well recognised opinion polls such as ‘Which?’ [18] consistently evidencing poor scores in consumer trust surveys with energy providers. There continues to be perceived poor customer care and lack of value for money.

The well-evidenced, related public health context is worrying. According to Public Health England [19], low income, high energy costs and the energy efficiency of the home are key factors in household fuel poverty. At the start of this project, in 2012, in England, there were 2.28 million fuel-poor households [20]. Fuel poverty can lead to cold homes. Whilst not all excessive winter deaths (EWDs) are caused by cold housing or low indoor temperatures, the World Health Organisation [21] estimates that between 30% and 50% of EWDs may be attributable to cold indoor temperatures. It was also calculated that at least 65 people a day may die in the UK in winter as a result of illnesses due to cold homes [22].

The majority of EWDs occur in people 75 years and older [23]. In terms of morbidity, for older people, cold temperatures increase risks of respiratory problems such as asthma and bronchitis; strokes and circulatory problems; hospital admissions and recovery issues following hospital discharge and also impacts on dexterity and lower strength that can increase the likelihood of falls and accidental injury [19],[24]–[27]Home temperatures can also impact negatively on mental health, for example cold is linked to anxiety and depression [28]. Cold homes are also known to have a negative impact on social activities, for example not wanting to invite friends into a cold house [29]. It is estimated that cold homes annually cost the NHS £1.36 billion [30].

In response, in 2015 and towards the end of our evaluation, the UK Government produced the first fuel poverty strategy in 13 years [20]. This followed an independent review of fuel poverty led by Professor Sir John Hills in 2011which concluded that it is unacceptable that people are prevented from achieving such warmth due to the combination of having a low income and living in a home that cannot be heated at reasonable cost. The strategy seeks to reduce bills and ensure comfort and well-being in the coldest, low income homes. More recently, in 2016, a jointly Government and charitable (National Energy Action) funded scheme, the ‘Big Energy Saving Network’, was launched to support eligible third sector organisations and community groups, to deliver help and advice about energy and switching to vulnerable consumers.

There is also national policy in place to address fuel poverty, for example The Energy Companies Obligation [31] was recently extended from April 2017 to September 2018 under its third obligation period. This is a UK government energy efficiency scheme to help reduce carbon emissions and tackle fuel poverty. This has been in place since the 2013 Home Heating Cost Reduction Obligation (HHCRO) obliged suppliers to promote measures which improve the ability of low income and vulnerable household to heat their homes and also encourage heating savings, such as repairing or replacing a boiler. There are also schemes directly supporting older people such as the annual winter fuel payments (between £100–£300) for those of pension credit age, the Cold Weather Payments of £25 payable to those on a low income when the temperature falls below 0°C for seven consecutive days [32].

However there is still cause for concern as England's fuel poverty statistics for 2018 (based on 2015 data) testify. In 2015, the proportion of households in fuel poverty in England was estimated at 11.0 per cent (approximately 2.50 million households). This is an increase of 0.4 per cent from 2014. At a time of an ageing population, continued austerity and financial cuts to services, more needs to be known about how older people manage their energy consumption and bills, and what knowledge and skills they may need to deal with rising fuel prices, address cold homes and, longer term, be able to have some financial security through affordable and flexible savings. Moreover to understand such high levels of customer dissatisfaction we need to access, listen and respond to local cultural narratives of their relationships, encounters and ongoing customer journey with the ‘big six’ energy suppliers.

## 3. Evaluation of the managing money energy advice programme

### 3.1. The project location

In the north east of England, Durham has a strong heritage of agriculture, and coal and steel making. Since the mines and steelworks were closed in the 1980s, the local population has been in decline, with younger people leaving and an increase in the percentage of older residents. Whilst many older people, including those living alone, experience independent and fulfilling lives, research shows that this group are more at risk of experiencing higher levels of disease and disability, lower physical activity, poorer diet, have worse memory and mood, be at risk of social isolation, have no emergency carer and may have reported and unreported falls [33],[34], among other problems.

Such age-related, potential problems are exacerbated by low income levels and also living in an area with dispersed settlements and very limited transport links. This in turn can result in lack of access to services and advice, adding to the problems of isolation and loneliness. The index of deprivation, measuring levels of deprivation across seven different domains of: income, employment, health, education, housing, crime and environment, indicates that County Durham remains in the top 30% most deprived authorities across England [35].

Localised engagement with residents identified the need for increased money management and money advice services for older people in the area. In 2013, a partnership led by a registered credit union and a registered social landlord, and including Local Authority, National Health Services and voluntary sector representation, successfully bid to a national Comic Relief programme, ‘Managing Money Better’, designed to support older people to cope with the challenges of the current harsh financial climate.

The service was based on expert Energy Advisors offering free home visits to:

- Support financially excluded older people unable to take advantage of lower fuel tariffs.
- Provide an at-home financial and energy consumption assessment.
- Improve money management skills.
- Provide practical support and help.

### 3.2. Qualitative evaluation

In addition to a quantitative evaluation focusing on financial savings, the authors were approached to carry out a qualitative evaluation that assessed the quality of life impacts of the energy advisor service on participants and also captured their approaches to energy use. A qualitative process evaluation was agreed, i.e. the research team walked alongside the project to collect data from different sources, to consider: how the ‘Managing Money Better’ (MMB) programme was being delivered, received and acted upon; and whether such activity was addressing identified objectives and meeting planned outcomes. Process evaluation enables the research team to address potential gaps in what may be planned and what is actually delivered, give explanation for such change and also, working with the programme partnership and in response to ongoing evaluation findings, seek to find ways to address any notable gaps. Northumbria University ongoing ethical approvals were also sought and granted with each new development.

### 3.3. Sample and recruitment

Recruitment was planned via the Energy Advisors who were supported by the credit union and who, through wide referral routes (e.g. GP, health and social care services, charities, housing agencies, word of mouth, self), were in touch with older people interested in receiving a home visit. As a qualitative study, we did not seek a representative sample, rather one which reflected the range of older people utilising the service and guided by the following inclusion / exclusion criteria:

- Inclusion: Age (50–74; 75 years and over)
- Inclusion: Gender
- Inclusion: Locality (urban/rural)
- Exclusion: Does not have the capacity to make an informed decision and give informed consent

Ethnicity and disability were also considered but not used because of the low number of ethnic minority older people in the area and the likelihood that a large proportion of older people in this area experience disability of some type. Overall, 34 participants consented and took part in a home or telephone interview or a focus group.

### 3.4. Data collection

Due to the evolving nature of the service being evaluated, a flexible approach was taken to data collection methods and timing. To gain the participants' perception of the MMB service, years one and two included in-home visits with the energy advisors, followed by a face to face interview; participant focus groups, telephone interviews and face to face interviews with the energy advisors. In year three, to analytically further explore the emerging findings, capture logistical and content changes to the service and better understand the individual contexts of the participants, a purposive sampling approach was used. This allowed participants to be selected on grounds of representing the key ranges of overall service delivery captured within service data collection records for a four-week period. Clients were selected from those having received an energy advisor home visit in January 2016 via a sampling matrix covering age, location and estimated financial savings made directly as a result of the energy advisor intervention (amount likely to be saved on energy costs per year {£0 = low, £1–200 = medium, £200+ = high}).

**Figure 1.**
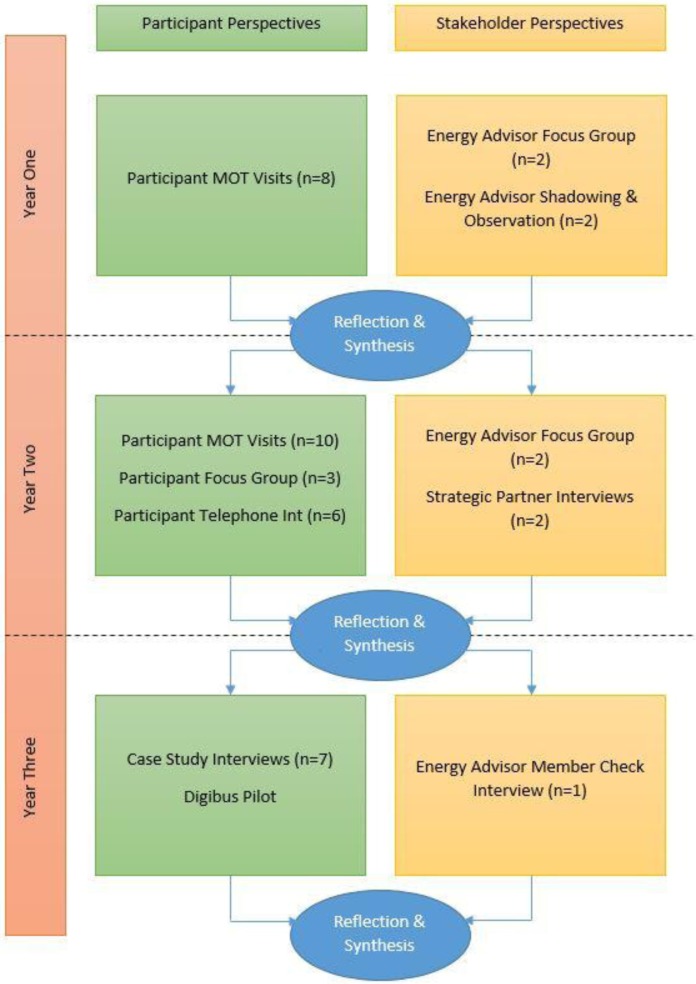
Data Collection.

Following each data collection period, findings were discussed amongst the project team to identify points at which data saturation had been reached and gaps where further consideration and focus was required. Four Energy Advisors consented to taking part in one-to-one interviews and/or a focus group. Within Year Two, and as a mid-way, quality assurance check of the process evaluation, the two overall strategic partners were interviewed to ensure that the process evaluation was focusing on Managing Money Better (MMB)'s overall objectives, whilst also being open to emerging findings. Figure 1, shows the range of methods and approaches to data collection used throughout the study.

### 3.5. Data analysis

The data consisted of the interview and focus group verbatim transcriptions. Inductive thematic analysis [36] guided the ongoing data analysis. Firstly, the data within and across each interview were grouped under similar codes. Codes were then compared each to the other and collapsed for further grouping of the codes into larger categories denoting further analysis. Categories were then critically questioned in relation to the data and to existing theory [37]. For example: ‘does this category make sense across a range of the data?’ ‘How does this category relate to what is known about this study area?’ In this way categories were further grouped, extended or challenged so that there was a set of themes both grounded in the data and theoretically useful.

### 3.6. Findings

Our qualitative approach emphasised capturing the stories and perspectives of the recipients (older people who received the ‘Managing Money Better’ service) and the providers (the trained Energy Advisors [EAs] and strategic partners) of the MMB service. To illustrate diversity of participant living circumstances, the issue that they wished to discuss with the Energy Advisor and outcomes, Table S1 presents short vignettes of 17 of the 34 participants. Findings are then presented thematically: *‘a front line service’; ‘bridging the supplier/customer gap’; ‘energy providers: ‘they're a secret society’; ‘better the devil you know’ and ‘digital exclusion and lack of control’.*

### 3.7. A frontline service

***Energy Advisors (EAs) described their role as frontline***, face-to-face contact that builds trust, so that the client shares their problems, issues and concerns. The advisor can give clear and **usable knowledge**, from how to interpret a utilities bill, to taking meter readings, to setting thermostats efficiently, to understanding how a gas boiler should be working.

**EA [1int.1]** “Nobody is born knowing how to read a gas bill. And it's just… And Mr A was particularly really good for me, because he just said, *“I'm so frustrated. Why are they charging me more?”* And I sat down with him and I said, “*This is what happens. This is how they look at your bill; this is why your direct debit has gone up.”* I think we actually got him a new boiler as well. [. . .] And just being able to sit down with the bills and that. [. . .] And he looked and… *“I don't know if I can do that?”* I said, “*We can. We'll do it together.”*

Another **key role for the EA is *making the initial telephone contact with the utilities company*** in order to query a sudden increase in a bill, a change in tariff, sometimes just to find out if there is a better tariff for the client. Within an interview with two of the advisors, they suggested and emphasized that 70% of older people whom they visit just need reassurance and face-to-face initial advocacy that then instils confidence for them to then ‘take control’:

“*We get a lot of*, ‘am I getting the best deal?’ ‘what's best next time?’ ‘Can I use my boiler or radiators better?’ *that sort of thing [. . .]. Sometimes you get someone [. . .] get how to read their bill or feel that next time, they can sort the meter reading, they don't need ongoing support although you can offer it.”*

However sometimes making telephone contact with an energy provider and getting accurate, usable and understandable knowledge is a challenge even for the experienced and expert EAs:

“To do something with the utility company, you've not only got that wait time, which could be an hour sometimes, when you get onto the phone you could be passed from one department to another. Or you could be answered with ‘the systems have gone down’. Or, you know… So sometimes it's what should be an easy process is a very difficult one. Unnecessarily.”

EAs also suggested that for some 30% of older people they have visited, the issues raised are complex, or they feel overwhelmed and a follow-up visit would be beneficial:

“It's just too much to take in and if they could have someone there, the first time they have to read the meter or work out the bill or phone the company, just someone giving them a bit of confidence, in the long run that'll save money and they will then teach others. The first meeting you just sow the seeds; you have to then look after them.”

Complex issues raised, ranged from a set of circumstances that had spiralled out of control such as insecure earnings (for example working with a zero hours contract or self employed) exacerbated by a period of illness, this leading to a change in welfare entitlements such as housing benefit and/or an increase in energy use), or illness leading to needing to heat the home during the day Such a change in circumstances often required form filling to avoid incurring benefit penalties (e.g. for not declaring a change in circumstances) that for some, was ‘just too much’ and difficult to navigate.

Energy Advisors reflected on what attributes they needed to be effective in a frontline role. One was ***having an established background in energy advice:***

“We're actually trained energy advisors. [. . .] We've always been involved with energy advice, whether it is on the phone or in people's houses. And previous to this, we've both worked for utility companies as well. So this is really… I would say our bread and butter. This is what we do.”

***They were also trained and accredited*** and needed ***intimate knowledge of the locality and its infrastructure***:

“We'd worked in the industry for, you know, a few years. [. . .] Which gives us a really good local knowledge as well. Which says a lot when you're working in... in that area. You know, we have an idea of what the houses are like. The housing associations, the communities, what's available, what's not available.”

And experience of working with **other agencies**:

“A lot of the time when we walk into their [client] house, there's bigger issues going on at the same time. So, you know, it's not only giving our own advice, it's... It's also referring to where you think they need to be.”

EAs also suggested that the personable and friendly advice given was seen as a positive way of engaging people:

“Never had such a rewarding job, I know what I'm doing makes a positive difference.”

**Participants also endorsed the EA frontline service.** Having a personal service from an EA who came to their home to both mediate and offer information; to share valuable knowledge about how to read meters, bills and thermostats or how to apply for entitlements, seemed to instil confidence and trust. The following is an extract from one of the participant group discussions:

RFg1 “... you've got that much on your mind going on and you've got appointments to think about. And you think, ‘Oh, you know, I just can't… [. . .] Well, I should be hassled because we… we can't afford it, you know. So someone actually coming in independent and saying’; ‘Right, okay, well I'm going to ring them up. And I'm not going to have any nonsense. I'm going to say to them no… You don't pay to get the meters changed. They can't afford to pay.’”

RFg3 “I think the fact that the EA is, and the other people they work with, they are independent. So they have a different… A different outlook on…”

RFg2 “. . . Well, they know how to talk to these people [utility company] on the phone.” [. . .]

RFg5 “And it's also because they're non-judgemental.”

### 3.8. Bridging the supplier/customer gap

That EAs were seen as non-judgemental or neutral, was perhaps pivotal to them being able to bridge the perceived supplier/customer gap, particularly when personal circumstances had overwhelmed the participants and they felt ‘pre-judged’ by their energy provider. A participant in his 50s described a set of personal circumstances, including a car crash 2–3 years prior to the interview that left him permanently disabled, a relationship that became acrimonious, issues with standing orders and very high energy and phone usage, which had resulted in escalating debt. The local credit union put him in touch with the EA service:

RFg3 “It was over £2000 [the fuel debt] and I'm permanently disabled [after a car crash] and I'd gone from standing orders to pre-paid meters and now they were threatening a key meter and I didn't want that. They own it and I really thought I could lose my home. It's mine, all I've got and then X (EA) came and [. . .] sorted everything, the debt's cleared, I have my home. You have no idea how the energy company treats you. It was always my fault, I was doing a scam, they were really aggressive and that makes you very vulnerable.”

Pre-paid meters (PPMs) can be installed in domestic homes and may be preferred by those on low or insecure earnings. They offer a ‘pay as you go’ tariff, where customers pay for their energy before they use it, usually by adding money to a ‘key’ or by purchasing special tokens. Utility companies may also insist on installing them within households where debt is mounting. How through use of a PPM, a large debt might accumulate and how a client may be unaware of the reason for such an accumulation of debt was explained by one of the EAs:

“Some people have got debt on the meter. And when it's not explained by the energy companies, they can put £10 on the meter and £7 is taken away straight away to cover the debt. And then added on to that, maybe £1.50 for the standing charge for the week. Which leaves them with £1.50 worth of electricity. Now we would, at that stage, get in touch with the energy company and get them to explain the circumstances. Get them to take the payback to be down to an absolute minimum.”

The Energy Advisor also explained how they can help clear a large debt:

“I went to a lady who was struggling with just her daily living. [Due to a large, unpaid debt], they [energy company] were due to come out and install the pre-payment meters, this lady had angina, COPD, she'd had an operation on her back. She was very upset.”

During the visit and with the participant, the EA completed a hardship application to a Trust fund that could support clearing the outstanding debt. She also stressed that there needed to be mediation with the utility company to ensure that there was a reasonable debt-recovery and prevention plan that was also in the best interest of the participant:

“…it was mediating with her and the utility company to say, “You can't… You can't just ignore it, you know. We need to set up some sort of payment arrangement and this is how I'm going to help you today.” But it was letting her know as well. We were going to do it together, so it's going to stop all these nasty bills coming through the door. And it's going to stop someone knocking at your door to install a meter than you don't want.”

Another participant described how during a time of dealing with her husband's cancer and his inability to continue with paid work, an unpaid energy bill escalated and subsequently became too difficult to tackle alone. A ‘good neighbour’ alerted her to the at-home EA service:

RFg1 “…at the time my husband was recovering from cancer, but he has had to stop work. And there's that much going on that, you know, you think, “Oh, I'll get round to it.” And you don't get round to it, you know. But if you know someone's coming and that they sit down with you then it's a lot easier, you know, to focus on it. They were very good.”

‘Bridging the gap’, mediation and being given invaluable information and advice was appreciated by participants, not just as support to deal with a crisis but also in terms of gaining new skills such as understanding a bill and being able to break down fuel use:

RFg2 “I mean, I do my own readings now and I know how… I could sit down tomorrow and work out how much I'm going to have to pay.”

As an EA recounted, clients also described feeling empowered to pass on useful knowledge to others, “And he took pride in sharing this knowledge with others, passing it on so's they've got a handle on it too”. Another EA however cautioned that acquiring and using this sort of knowledge may not be easy for those who might feel that, “well, they're in a mess because they can't manage”. Another suggested: “It's a pride thing with people. And I think a lot of people wouldn't want to come forward and go, like… Because just out of pride.”

It could be said that there were competing narratives in terms of being a ‘capable citizen’ who manages their affairs, even in very difficult circumstances, but also a shared belief that utility companies are not good at passing on critical information, such as how pre-paid meters collect debt or how they estimate a bill when unable to access a meter. Certainly in terms of the latter, we listened to many stories of mistrusting energy providers, an extreme view being that they are like a ‘secret society’.

### 3.9. Energy providers: they're a ‘secret society’

Energy Advisors spoke of some clients being very suspicious of their role, perhaps because of their wider-held beliefs that energy providers in general are deliberately misleading, “out to scam you”:

“Some of the older people, they have someone with them. I've been to a few properties where family members can be very suspicious: ‘who are you employed by?’; ‘what is/are your company/you getting out of this?’ They think there must be a commission and that's why we always introduce the project as funded by Comic Relief with trusted partners such as [social landlord] and Durham County Council, that's important, trusted brands.”

As the free at-home MMB, energy advice service became embedded within local communities, the notion of a ‘trusted brand’ developed around this service. An extract from one of the Year Three case studies illustrates this:

“This participant had a long-standing distrust of energy companies who he felt were ‘conning people’. He had previously had difficulties with customer service and changing to cheaper tariffs. He heard about the service via a friend, and was impressed by the rumoured savings to be had and the approachability and friendliness of the EAs. Wanting to save money, a visit was arranged. The advice given, both on energy suppliers and use, was given as helpful education, rather than enforced instruction, encouraging him to successfully change supplier and make a yearly saving.”

On the whole, however, attitudes towards energy suppliers were negative. Participants did not trust shared information, found accessing and understanding information difficult and some reported feeling stupid and that the energy provider refused to take responsibility:

RFg5 “…It's blame, punitive culture. So it's almost like they're waiting for you to do something, it's your fault, but when they do something [wrong] it's not their fault.”

There were also intimations of anxiety and worry caused by what seemed to be an intractable or ‘out of my control’, issue with the energy provider but which was addressed through the MMB energy advisor ‘stepping in’, offering support and alleviating the worry:

Why would somebody who owns a house, who's paid for the house, who's crippled, who never wants to move—why would I do this to myself? Right? But it took [EA] getting on board. And I just found that I actually didn't believe until she walked into the room, because of the type of personality and sort of aura she's got, I actually had faith in her from when she turned up. But I didn't actually think she would get it all sorted out by… So anyway, the conclusion for me is the only other thing I can say about it is that it saved me from having to sell the house, which is now the last thing that I've got.

Another focus group participant also suggested that their encounter with their energy provider left them feeling vulnerable:

RFg2 … “But the worst part is that [Energy Company] have a vulnerable person's unit Well, it makes you more vulnerable if you get in touch with them. And I actually got in touch with all this because it actually mentally broke me. [. . .]

Whilst in the meantime they go… They conveniently go, In the meantime, ‘would you like to have this key meter temporarily.’ And if you're stupid enough to say yes, you're stuck with it forever.”

Others were frustrated with the inflexibility of a service that could not be tailored to changing need. Whilst most participants were well aware that direct debit is cheaper than a prepayment meter, for those on insecure income (e.g. benefits may change, uncertainty about employment; worry about not having ‘spare funds’ should there be an emergency), the former is not an option. For these participants there were expressions of ‘living on the edge of society’, ‘being discriminated against and unfairly’ and being ‘forgotten’ or ignored’.

Also, as this extract from one of the participant discussion groups illustrates, for some participants dealing with the utility company's ‘paperwork’ was stressful and asking for help was not always easy:

RFg3 “But when we look at my Mam, who was nearly 88, there is no way on this earth that she could understand the paperwork coming out [from the energy companies]. {. . .}. And it was really a sigh of relief when [EA] stepped in and sorted it out. Because… My ma kept a lot to herself as well. You know, and I think older people tend…”

RFg4 “They don't bother.”

RFg2 “Yeah, they don't want to bother you, do they?”

RFg3 “They don't want to bother people in case you think that you're annoying them or, you know, whatever.”

RFg2 “And the energy companies, when you ring them, it was… because we, at one time, had pre-payment meters. And they just kept saying… yeah, they just kept saying, ‘Oh, you know, you have to pay the balance off’, which was something like £40 or something stupid. You know, but they didn't give you any advice. They just, sort of, left you to sink or swim, you know.”

“Better the Devil you know.”

Not all participants were willing to switch providers or tariff, even when they knew they could make a saving. This was often due to distrust over certain tariffs, particularly those only available via online billing. For others though, it seemed to be a case of “better the devil you know”, sticking with suppliers and methods of payments they knew and trusted. This level of control was particularly found in relation to pre-payment meters which, despite being more expensive, were often viewed by those who had them as offering a level of personalized control that was reassuring. As an energy advisor commented:

“…They just like the fact that, you know, they know if they put £10 on and they run out, they're just… They're just more happy in doing without gas and electric than having a huge bill come.”

Even if there are challenging issues, clients seemed reluctant to switch energy company. People trust what they know. If they pay bills weekly through a Post Office account, or rely on a good friend/neighbour to help with money management or have always used pre-paid meters, they don't seem to wish to consider other options, even if they are financially beneficial. However they also needed time, to consider, to act, to change behaviour. As one of our case studies highlight, an unexpected change in circumstances, with timely useful advice and information imparted with clear emphasis on needing time to make a decision that ‘going against the grain’ may seem risky, can act as a catalyst to change expectation, or an accepted cultural understanding of how such a service might operate:

“The participant was recently forced to retire due to a change in health (developing COPD). With a considerable reduction in income, he was referred to the [EA] service by his GP. He already considered himself knowledgeable about his energy costs and use, but had never considered the need to change until his current change in circumstances. He found the EA to be very helpful [...] he was initially reluctant to change supplier as he was wary of potential costs incurred on moving contract, but was able to make a substantial saving when the EA took him through the process [...]. He has since challenged several other existing contracts, and negotiated cheaper deals for his television and mobile phone packages.”

### 3.10. Digital exclusion and a lack of control

Not switching or changing tariff was sometimes to do with reticence about using technology as some of the cheaper tariffs are only offered online. As a result the EAs suggested that some participants ‘miss out’ on online billing and ‘getting the best deals’ and, in one of their discussion groups, there was a suggestion that this was to do with age:

EA1 “Not being ageist but some of the older customers, well some of the younger ones too, but thinking about the older ones, there's two things, they might not have used computers at work, family might do things for them now, they might think it's an expense and getting to a library round here [rural communities] can be expensive too [public transport], so getting them to take on online billing and talking about the online deals doesn't happen. I think it will change, different generations will be more computer savvy but perhaps there needs to be some support now, some online support for these people.”

EA2 “Yes but also older people, usually, they don't like change, they like to stick to what they know…”

EA1 “…but well yes but that's not just older people, most of us don't like change [laughs].”

(Year Two, Focus group)

Our participant age range (50 to 80+) spanned at least two generations and from within our case studies (these purposively sampled to illustrate the diversity of participant profile), the majority were in the 60–69 age bracket. Overall these participants described online services as the cheapest option and most convenient for monitoring energy expenditure and changes to billing and tariffs. For those not using online services, there was expressed reluctance to change and a suspicion of energy providers, particularly via the belief that direct debit payments could be changed without the customer's knowledge or approval. Thus, despite the benefits being reported elsewhere by participants, in this case the lack of use was dependent on a broader view of such services which may be difficult for the advisor themselves to alter.

Despite the efforts of many EAs, there was difficulty in getting those accessing the service to change their approach to online billing. Whilst some participants suggested that “computers were never my thing and I ‘m not starting now”; “ you can get free computer lessons through [social housing] but you don't get any follow up and I don't have my own computer”; “ the granddaughter is a ‘divil’ [devil], she'd put god knows what on one of them things (PCs) if I had one and I'd be paying”; overall reluctance about online billing was also to do with suspicions surrounding a perceived lack of control of direct debits and fears of unexpected costs contributed to people being unwilling to change their approach.

## 4. Discussion

Our qualitative process evaluation of the three-year UK Comic Relief-funded, Durham-based, energy services and Managing Money Better programme, set out to assess the quality of life impacts of the energy advisor service on participants and also capture their approaches to energy use. Our findings point to positive impacts, including reducing high anxiety about unmanageable bills, being able to question and challenge energy providers' ‘runaway’ bills and tariffs, acquiring new knowledge and skills such as how to read a utilities bill and passing on such skills to others. The ‘frontline’ MMB, EAs, those offering the free at-home service, also emphasized that some 70% of older people whom they visit just need reassurance and face-to-face initial advocacy that then instils confidence for them to ‘take control’ of their energy consumption and expenditure.

We also suggest that by listening to both older person and energy advisor, telling of encounters and dealings with the energy companies, we were made aware of ubiquitous local cultural narratives, arising from personal, negative, customer experiences with energy providers. Whilst some participants recounted ‘facilitative’ narratives about their dealings with energy providers, including friendly service, efficient and effective handling of a query, there were far more recollections of perceived, negative communication. Echoing Davidson [5], it seemed that first hand stories of a ‘rigid’ energy supplier with inflexible penalties resulting in out of control bills, became others, second hand ‘narrative truth’, i.e. energy providers are punitive and treat their customers as reckless, regardless of anything in between. While there was evidence of the participants holding these cultural understandings, it was also noticeable how they were often reinforced by ingrained practices within the energy provider service, for example the distrust of the supplier was often reinforced by the complexity of bills making them difficult to understand or long waits when telephoning with queries or requests for further information. For us, such cultural narratives raise three issues that we need to address if public good is to come from public health research.

Firstly, in relation to engendering a mutually beneficial customer and energy supplier relationship invested in trust, we need to consider addressing, potential discordant ‘ways of knowing’, through narrative change. We need a shift from the paradigmatic way of knowing via verifiable facts and empirical truisms [2]. Instead, narratives which capture culturally-specific and complex social constructions and meanings ascribed to public health matters, could be more beneficial for customers. Such different ways of knowing seem to entrench mutual distrust. Equally, when this is reinforced by a clear power imbalance, in which the failure to understand and act upon the given empirical truth within a different cultural narrative, results in penalties, such as late payments fines, the negative relationship is further exacerbated. By acting within a face-to-face frontline service, the MMB EAs were able to negotiate and influence participants' cultural narratives. Their advice was more appropriate, accessible and effective as it could be understood by and negotiated with, the participants.

Secondly, this becomes even more clear when examining in detail how the EA role differed from that of the energy providers. EAs holistic model of energy provision, created a situated social, cultural and economic understanding of the customers multiple perspectives. The EA began with local and individual perceptions (knowledge, beliefs, attitudes and motivation), rather than ‘need’ as identified by the experts (the providers), to consider what actually was available to customers in this context. This was reflected by the extent to which customers passed on and shared messages via word of mouth, both individually and collectively. This holistic approach fits well with socio-ecological models of public health behaviour change [10].

Thirdly EAs invested in forming trusted relationships with their ‘customers’. Investment included visiting people in their homes, behaving in a personable way, being a named face-to-face contact and sharing facilitative information that enabled the individual to make an informed choice and maintain a sense of dignity, control and power. EAs were perceived as effective in offering an alternative relationship to that of the impersonal and faceless procedures of the energy providers. As such, they were able to bridge the gap in the narrative of ‘them and us’ and were perceived as very much ‘one of us’, but with the benefits of the valuable understanding of how’ they [the energy providers] work’. The face to face EA service became a ‘trusted brand’ and in and of itself, provided a space for a new narrative [5], or narrative change. Narrative then, can be an influencing tool, in this case, challenging the perceived, dominant, rational and ‘dehumanised’ energy provider model of ‘customer care’, to one nuanced by situated social, cultural and economic understanding of multiple perspectives [1]. The new narrative though has to ‘ring true’ and this takes time to then become trusted.

Overall we argue that to address the perceived imbalance and inequity between energy providers and their ‘customers’, further understanding is needed of how cultural narratives influence behaviour and how they are rooted in ‘socially learned’ distrust. Even without personal, individual experience, such narratives may become collective givens, the ‘word on the street’ about what to expect from a service or provider. The current narratives have created strong oppositions, positioning ‘rigid and profit hungry suppliers’ on one side and the ‘vulnerable and uninformed customers’ on the other. From a public health context this may exacerbate fuel poverty and cold homes. The face-to-face energy advice service that we evaluated and reported here, bridged these oppositions, by acknowledging and understanding the cultural narratives on both sides. Where this isn't possible, support may be required to ensure that energy suppliers understand the importance of adapting to these narratives. Without such a cultural shift, energy services that fail to acknowledge the entitlement of access to energy and provision tailored to changing circumstances, may continue to deepen these oppositions.

## 5. Limitations

Participants were identified by the Energy Advisor team, rather than by the evaluation team. Although this means that it remained difficult to ensure participants reflected a wide range of those in receipt of the service, the use of the EA as a point of contact and information about the study for both the researchers and participants was invaluable in accessing potential participants. During home visit data collection, the evaluation team was scheduled to accompany the EA during appointments pre-booked by the EA team, so that data collection was carried out during a ‘routine’ day. Also our cultural narrative lens emerged from the evaluation and focused very much on both the perspective of the ‘Managing Money Better’, energy advisor team and our participants, the service users, supported by the MMB ‘front line’ service. We concede future research should also include capturing the experiences of the energy provider, front line workers, particularly those manning the customer care and help-line phone communication. This would also enable the exploration of local cultural narratives from ‘provider’ as well as ‘receiver’ perspective.

## 6. Conclusion

Most of us in developed countries such as the UK, rely on energy providers to supply essential energy services such as gas and electricity. These being affordable, accessible and secure, are vital to our physical and mental health. It seems reasonable then to assume that it is mutually beneficial to have a customer and supplier relationship invested in trust. However certainly in the UK, there appears to be a dominant, negative cultural narrative about energy providers and their customers not trusting each other. Our findings from a qualitative evaluation of the three year UK Comic Relief-funded, energy services and ‘Managing Money Better’ programme, seem to bear this out. However the MMB, face to face EA service, has the potential to bridge the ‘two sides’ and open up the possibility of narrative change, a change that could materialise in mutual and flexible understanding of the lived context of using and paying for an energy service, that in turn, should foster workable trust. For public good to come from public health research, we need to understand and appropriately address, situated and embedded narratives from both ‘user’ and provider side.
