# Supplementary material for: On the Defence: UK cultural narratives of mistrust between energy users and providers
Source: AIMS Public Health. 2018 Mar 13;5(1):31–48. doi: 10.3934/publichealth.2018.1.31 (PMC6070460; doi:10.3934/publichealth.2018.1.31)
Supplement: Supplementary file 1 [file publichealth-05-01-031-s001.pdf]

Public Health

AIMS Public Health, 5(1): 31–48.
DOI: 10.3934/publichealth.2018.1.31
Received: 11 July 2017
Accepted: 10 January 2018
Published: 13 March 2018

http://www.aimspress.com/journal/aimsph

Research article

On the Defence: UK cultural narratives of mistrust between energy users and providers

Cathy Bailey* and Philip Hodgson*

Health and Life Sciences, Northumbria University, Newcastle upon Tyne, NE7 7XA, UK

* **Correspondence:** Email: Catherine.bailey@northumbria.ac.uk, philip2.hodgson@northumbria.ac.uk; Tel: 44(0)1912156224.

Supplementary

Table S1. Participant breakdown, Years one and two.

Profile	Background and Query	Outcome
**78 years, F**<br><br>**Social renting, 2 bed bungalow, living alone**	Slightly deaf, but mobile and independent. 17yrs tenancy. 3 years previously, landlord refurbished home, including energy efficiency features (e.g. insulation; double gazing). **Query with boiler and standard rate utility tariff**.	EA phoned utility company, **fixed rate tariff for 2 years agreed with client; issue with boiler is being addressed by landlord but EA will also send on query**. Client has bank account, pays by direct debit and did not wish to join community bank, or need benefit advice – **very happy with tariff change**
**70 years, F**<br><br>**Social renting, 2 bed semi-detached, living alone**	Widowed for 3 years, very dependent on family to get out as impaired mobility due to arthritis, says she is quite lonely and son and grandchildren moved to Canada 5 years ago. Home is insulated; double glazed, gas central heating. Says she doesn't feel the cold, even though sitting quite a lot and keeps heating at about 17 **Query if she can reduce utility bill but text in document suggest she is on cheapest rate**	EA checks utility document with client and suggests standard rate (this is variable) is not cheapest rate (fixed term, non-variable usually is) but that text is ambivalent and offers to telephone utilities company to query**- unable to get through. Client will call stating the EA will be making contact on her behalf**.<br><br>**EA also says that heating (boiler) will work efficiently (value for money) up to 20**<br><br>Client pays by direct debit, **wants to stay with this company, feels that she has all benefits entitled to and does not wish to join the community bank**

Continued on next page

Profile	Background and Query	Outcome
**54 years, M**<br><br>**Social renting, one bedroom, living alone**	Living in property 18months, has disability allowance, some eyesight problems, daughter and son live locally (toddler granddaughter was with him). Did not have direct debit, using payment card *"was higgledy piggledy, sorting benefits, so just carried on with the card"* and have switched energy provider *"couldn't understand all the paper work"* & now not with one of the big 6.Mentioned some damp in the home despite insulation and double glazing **General query about 'best deal'**	EA unable to calculate annual consumption as client switched companies within 18 months but did note **client received estimated reading & offered meter reading support so that client could avoid estimated reading bill**<br><br>Also that client **kept heating at 25, information about significant increase on bill for each unit over 20**<br><br>Client **not receiving 'Warm Homes' 'discount but Client's company not one of the 'big 6'**, so not entitled. EA contacted energy comparison site to query better deal for client if he switched to one of the big 6 – **preferred to stay with present company for now**<br><br>Client did say that with benefits now sorted, **would consider direct debit payment as cheaper than payment card**<br>**EA also referred client to welfare rights team and filled in enquiry form for COMMUNNITY BANK to follow up**<br>**Client very happy with all the information**, "*I feel a bit more in control, it's hard to take it all in with everything else going on"* and was reassured that the referrals would be followed up

Continued on next page

Profile	Background and Query	Outcome
**78 years, M**<br><br>**Social renting, 3 bed, semi-detached, living alone**	Smoker living with a small dog. Pays bills quarterly in cash. Has a bank account *"but I like to see what I'm paying and go and pay it over the counter*". During the visit he couldn't find his bill, identified his thermostat but didn't understand about the setting. He thought his boiler was guaranteed and in good condition. He was unclear about whether he was on a standard or fixed tariff but transpired that his energy provider had recently contacted to offer another fixed rate 2-year tariff as existing one was about to expire. **General query about energy bills and possible savings**	EA ascertained that client was on disability benefits and that he was unclear about entitlement to energy specific benefits; **she phoned his energy company and registered him for the 'Warm Homes' annual £140 reduction to winter fuel bill**<br><br>**EA also explained thermostat setting**, checking it and saying that at 20 this was fine but explaining that "for every one degree over, you pay 10% more on your fuel bill"<br><br>Whilst client couldn't find his paperwork, conversation suggested that **energy provider was sorting a further 2 year fixed tariff and client was waiting for confirmation paper work. EA suggested that client could make contact with her it there were any queries**<br><br>Client was very happy with the visit
**69 years, F**<br><br>**Social renting, 3 bed semi-detached, living alone**	Living alone, met in neighbour's house as looking after 'nervous dog'. Has problem with damp in utility room that council landlord will address but client wishes to settle dog first. **Query about recent change in tariff:** *"they did send some information offering me different tariffs. But [. . .], they could bamboozle me with science, you know."*	**EA stressed to client that she should address damp as priority for fear of it spreading, having detrimental impact on health**. Client agreed. Also delighted that this service is free and supported by charity and COMMUNNITY BANK. Keeps thermostat at 19 *"I hate heat me"*, received an estimated bill recently and *'corrected it'* so is able to read her meter. **Prefers to pay weekly via the Post Office. Standard tariff but guaranteed rate for 2 years – EA phoned provider and client very happy with this**<br>***Declined COMMUNNITY BANK services as friend (present) of 25 years sorts all finances for her***

Continued on next page

Profile	Background and Query	Outcome
**65 years, M**<br><br>**Social renting, living with partner, who had a brain haemorrhage 18month ago so now full time carer. 2 bed, semi-detached house**	Uses pre-payment meter (PPM): it's easier for us, really. Because we control it more, Query: need heating 24/7 as wife gets cold in the night , gas central heating , unsure if they can reduce bills	**EA explains that timer set to 20 and coming on for set hours, would save some money -** at present client just switching heating on and off: "*but she gets so cold in the night, she needs it"*; **Also PPM is more expensive than direct debit but client wishes to keep this system**.<br>EA phones provider and they are **launching fixed term tariff in the summer for PPM clients and asks for client to be put on mailing list**;<br>**EA gets client on priority register**<br>(if there is a break in service such as gas leak etc., company phones those on this list to sort alternative arrangements)
**87 years, M**<br><br>**Social renting, 2 bedroom terrace, living alone**	Daughter who lives locally had used the MMB services and her mother is coming to the end of a fixed tariff and wanted to consult with the MMB team. Client has 2 sons and 2 daughters living locally, she is a bit deaf, but mobile and likes to *"keep on top of things"*	Client is a low user but **switches her gas central heating on and off. EA suggests demonstrating how to set a timer for usage with some savings but client declines.**<br>Also she keeps a radiator on in her spare bedroom, although rarely used "*but my son, he might stay for a couple of nights and I never know when he's coming so I just keep it on"*<br>It is **too soon to be enquiring about changing fixed tariff** – usually this happens within 4 weeks of the existing tariff – **earlier switches could incur a £50 fee** so client decides to make contact with EA in August *"I hate change so will be good to do this with you"* and is very happy with MMB service

Continued on next page

Profile	Background and Query	Outcome
**64 years, F**<br><br>**Owner occupier**<br>**2 bed, mid terrace, old property living alone**	Good family support just retired and **query if using energy well as likes to be warm worried about how she will manage.** Home has no loft insulation, but does have double glazing, gas central heating and a log burner, uses thermostat and programmer for heating, currently on a fixed price deal no issues with this has switched before.	EA checked utility bills and advised on how to work out bill, **new readings taken and submitted by the EA on her behalf. EA made referral for loft insulation. Advice on using radiators and how to be more efficient with use of eclectic. Advice to try U switch in June.**<br>EA left contact details if client needed further advice. Information given on PB lady was aware of the credit union and reported that others found it helpful.
**60 years, M**<br><br>**Owner occupied mid terrace, 3 bed 1920s lives with wife who is on DLA**	**Just want to see if they could save any energy costs.**<br>Have good family support normally out at work during the day but currently of work due to surgery, have had insulation and cavity walls filled. Gas central heating. Use a thermostat to control use feel happy with current supplier.	**EA reviewed a current bill with the client and with the client's agreement rang the provider to check tariff**. Reassured the client that their consumption was below average which they were pleased about.<br>Offered advice on use of electric including the kettle and other appliances.<br>Advice they could get a cheaper tariff but couple wanted to leave as happy with current provider.<br>**Offered advice for wife on her review for DLA referral made to welfare rights for a review**
**65 years, F**<br><br>**Social renting, lives alone, semi, been in 11/2 years**	Would like energy review as thinking of retirement	EA gave advice on current use reassurance that doing really well. Discussed possible **savings from paying DD** client likes to pay by bill. **Reviewed the current bills** standing charge is high client tied in until April then need to review. Advice offered on benefit checks and referral for support. Client has good social support knows about COMMUNNITY BANK.

Continued on next page

Profile	Background and Query	Outcome
**60+ years, F**<br><br>**Home owner, single, 3 bed 15-year-old house**	Contacted by letter and though it would be good to have a review **wanted advice on how to use heating better**	EA gave advice on project. Discussed loft insulation client has mice in roof trying to sort out advised that the council won't do loft with mice but **referral made for a loft survey.** Advice re turning heating down and use of the thermostat keep it below 21 if possible. Current tariff good advice to review regularly rang through meter readings. **Discussed warm homes discount** may just qualify lady was going to review her finances to check EA will telephone to follow up. Advice on the credit union given.
**64 years, M**<br><br>**Social rented, single 1 bed bungalow 1950**	**Wanted advice on heating if using correctly**	**Uses pre-paid meters and likes this method**. Advice on warm homes EA reviewed bills and has claimed this. Reviewed bills and usage low. **No real needs identified**. Discussed COMMUNNITY BANK and client will be in touch as want a new sofa
**65+ years M/F**<br><br>**Social rented, 2 bed, bungalow, insulated central heating**	**Got letter and thought be good to review use**	**EA reviewed bills advised on a good rate, usage very low for both electric and gas.** Discussed possible energy savings by turning down radiators not having thermostat above 20. Advice about COMMUNNITY BANK and its services was offered. **Couple happy with support from EA** and will refer service to other friends.

Continued on next page

Profile	Background and Query	Outcome
**57 years, M**<br><br>**Social rented end terrace 3 bedrooms (one v small)**	4 yr. occupancy, living with wife and 13yr old daughter, diabetic, uses a walking stick, on sick leave, living room was v cold (thermostat at 5 – says he can't afford to heat home	**EA ascertained participant on lowest level of disability allowance and diabetic suggested that house was far too cold and needed to increase thermostat to at least 17-20**<br>**EA wished to secure warm home discount (£140) – but on phoning the energy provider found that there was an error in** their records and they had incorrectly inputted participant's name – participant would have to complete a form to undo this error – EA offered to do this. Participant's wife was also listed on the account but could not take the query forward without proof of correct name<br>**EA reassured participant and said she would follow with is through as matter of urgency,** she advised not to switch on the electric fire (this was done to 'take the chill off the room') but rather to maintain an ambient temperature with the thermostat
**80 years, F**<br><br>**Social rented end house, 2-bedroom bungalow**	Lives alone, husband had been invalid for 30 years, primary carer, and also nursed brother with dementia. Has 2 married daughters and grandchildren	**Received v high gas bill and worried about putting the heating on,** couldn't understand why it was such a big bill. Discussion about use of thermostat and regulating radiators – participant didn't know about latter and was grateful for information. Spends more time at home and wants 'peace of mind'. Energy Advisor read the gas meter and calculated 'huge' **over=estimate on gas bill – Participant coming to end of her tariff shortly but wished to change now and pay the leaving fee (she thought it was £30 but £5 was agreed) Energy Advisor efficiently called Switch to get the best deal and then another call to put this in place Participant was very relieved and grateful**

Continued on next page

Profile	Background and Query	Outcome
**74 years, F**<br><br>**Social rented mid terrace 2-bedroom bungalow**	Lives alone, recently moved in , just over a year occupancy, living with cancer ('I feel the cold')	**Doesn't like to pay by direct debit as at previous home, 'had horrendous trouble with energy provider' being over charged and not getting it back so now 'I just pay at the cash machine when the bill comes through' - this was a very traumatic experience – 'they sent threatening letters'; 'they were going to blacklist me' – 'and it went on and on.** Energy Advisor very sympathetic – suggested that current bill is too high and offered to find cheaper tariff which she did and participant v grateful also suggested turning down thermostat from 21 to 20 to reduce costs
**60+ years, M**<br><br>**Social rented**	**Lives with wife, just moved back last few months from south west, working on caravan site, originally form NE, one daughter settled in the States and a son living locally**	**Had very recently had pre-paid meters removed, welcomed support of Energy Advisor to set up tariff with energy provider on new meters,** could not instigate for further 4 weeks (old PPM needed final reading) but suggested direct debit and that Energy Advisor would support setting up new account. Also as claimant on unemployment benefit said they would be eligible for Warm Homes discount £140 Credit Union also discussed but participant not interested

AIMS Press © 2018 the Author(s), licensee AIMS Press. This is an open access article distributed under the terms of the Creative Commons Attribution License (http://creativecommons.org/licenses/by/4.0)
